# Avifauna diversity in the environmentally sensitive area: Alpha and Beta analyses in Kluang Forest Reserve, southern Peninsular Malaysia

**DOI:** 10.3897/BDJ.12.e137367

**Published:** 2024-11-18

**Authors:** Nur Aina Amira Mahyudin, Nur Athirah Fauzi, Kaviarasu Munian

**Affiliations:** 1 Zoology Branch, Forest Biodiversity Division, Forest Research Institute Malaysia (FRIM), 52090, Kepong, Selangor, Malaysia Zoology Branch, Forest Biodiversity Division, Forest Research Institute Malaysia (FRIM) 52090, Kepong, Selangor Malaysia; 2 Environmental Management and Conservation Research Unit (eNCORe), Faculty of Applied Sciences and Technology, Universiti Tun Hussein Onn Malaysia (Pagoh Campus), 84600 Muar, Johor, Malaysia Environmental Management and Conservation Research Unit (eNCORe), Faculty of Applied Sciences and Technology, Universiti Tun Hussein Onn Malaysia (Pagoh Campus) 84600 Muar, Johor Malaysia

**Keywords:** alpha diversity, beta diversity, bird, Environmentally Sensitive Areas (ESAs), Kluang Forest Reserve, Johor

## Abstract

The Permanent Forest Reserve (PFR) is recognised as one of the Environmentally Sensitive Areas (ESAs) in Malaysia`s spatial planning. Incorporating biological factors alongside existing physical attributes is crucial to improve the classification of ESAs. It is important to attain biological diversity information to formulate biological factors, which include vertebrates such as bird communities. Birds are highly sensitive to changes within ecosystems; hence, they play a pivotal role in reflecting the overall ecological condition. Therefore, the study focused on assessing bird species’ richness in the Kluang Forest Reserve (Kluang FR) and compared its bird diversity amongst five PFRs ESAs in southern Peninsular Malaysia. Methods such as mist netting, point count observations and call recording were deployed to calculate the alpha and beta diversity of the bird community. A total of 68 species comprised of 36 families were recorded and the white-rumped shama (*Copsychusmalabaricus*) was the most abundant species in all reserves studied. Principal Coordinates Analysis (PCoA) revealed that the bird diversity in Kluang FR is more similar to bird santuary Panti Forest Reserve compared to the other four forest reserves and indicates that diversification of species richness. However, the absence of published species information in forest reserves poses challenges for comparing bird assemblages amongst different reserves. More extensive studies are needed in Johor and throughout Malaysia to provide data that can effectively assist and support biodiversity conservation and management.

## Introduction

The Town and Country Planning Department of Malaysia (PLANMalaysia) has developed a comprehensive guideline on the conservation and development of environmentally sensitive areas (ESAs), which focuses on three crucial aspects: heritage value, elements of disaster risk and life support systems (PLANMalaysia 2017). Nine integrated management initiatives were identified within the guideline: conservation and development of coastal beach areas; water catchment areas and groundwater resources; floodplains, wetlands, mines, lakes and rivers; mineral deposits and geological disasters; landfills and solid waste disposal sites; agriculture and food; wildlife habitats; Permanent Forest Reserves (PFRs); and cultural and natural heritage. This guideline is applied during the preparation of spatial development plans in each State in Malaysia, with the goal of effectively managing and controlling the designated ESAs.

Amongst the identified integrated management areas including the PFRs, cover 54.58% of the land in Malaysia (Ministry of Natural Resources, Environment and Climate Change 2023), serving as major habitats for various floral and faunal species in the country. However, the classification of PFRs in the ESA guideline predominantly relies on physical attributes, such as slope gradient, elevation and risk level. Hence, the guidelines must be improved given their lack of including the biological factors that should be considered for ESA classification in PFRs. Shahfiz et al. (2021) proposed a new ESA classification approach for PFRs, incorporating biological parameters with the existing physical factors to reclassify the current ESAs within the country. They underscored the importance of documenting the biological data in various forest reserves to determine sensitive, precise and comprehensive biological parameters that should be incorporated into the current guidelines.

Shahfiz et al. (2021) proposed the documentation of avifauna as one of these biological parameters, which comprises quantifying the species diversity, the origin of the species and their conservation status, as well as the trophic composition. Biological factors play a crucial part in these ecosystems as demonstrated by the roles of birds in forests as bioindicators of the forest condition, as scavengers of carcasses and as part of the nutrient cycle by performing pest control and acting as pollinators and seed dispersers (Nason 1992, Zakaria et al. 2005, Sekercioglu 2006, Whelan et al. 2008). The presence of birds in various ecosystems underscores the importance of protecting and ensuring the proper management of each of these ecosystems. The information collected regarding these biological factors can provide insight into the overall health and functioning of the ecosystems within a PFR, help with assessing ecological balance and aid in identifying any potential threats. Therefore, documenting avifauna is essential for the incorporation of new biological parameters into the existing guidelines for ESA classification.

The State of Johor, comprising 23% forest, with 17% classified as PFRs, displays remarkable biodiversity despite its geographical isolation (Jabatan Perhutanan Negeri Johor 2023). Approximately 599 bird species have been recorded in the State of Johor (Avibase 2023). The notable bird hotspots in Johor, such as Tanjung Piai, Parit Jawa and Panti Forest Reserve, contribute to its status as an important bird area (IBA) (Lau et al. 2012). Panti Forest Reserve, recognised for its bird sanctuary, is a key bird location. However, the avifauna richness in Kluang Forest Reserve, particularly around Gunung Belumut Eco Park, remains underdocumented, emphasising the need for surveys and observations to increase our understanding of the bird species present.

In this study, the birds in the Kluang Forest Reserve (Kluang FR) were documented by the Zoology Branch of Forest Research Institute Malaysia (FRIM) from March to July 2022. The primary objectives of this study were to assess bird species richness in the Kluang FR and to compare the differences in bird populations amongst five selected forest reserves classified as ESAs in the southern part of Peninsular Malaysia.

## Material and methods

### Study site

**Figure 1.**
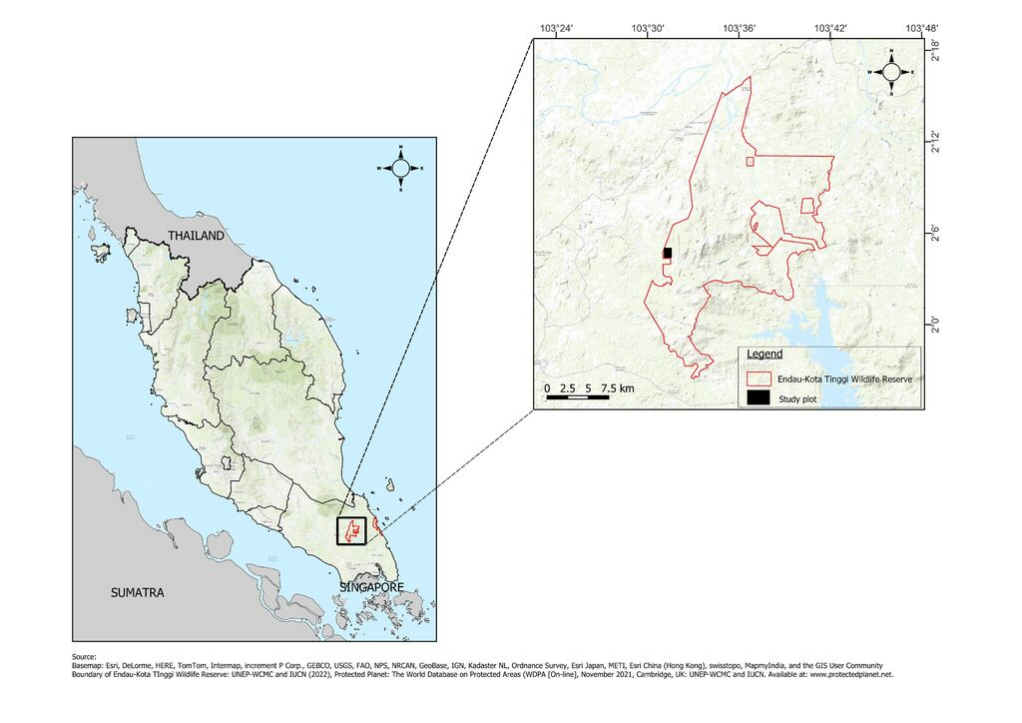
Location of Kluang Forest Reserve in the State of Johor.

This study was conducted in the southern region of Peninsular Malaysia, specifically in the State of Johor. Gunung Belumut Eco Park is located within the Kluang (Fig. 1), having an area of approximately 30,000 ha and primarily consisting of hills and lower montane forest. The highest peak is approximately 1,010 m above sea level, making it the highest-altitude forest in the state. The name ‘Belumut’ originated from the slippery surrounding and rocks covered with moss (Peh et al. 2005, Lim et al. 2012, Yahya Mahmood et al. 2012). Geographically, Gunung Belumut Eco Park mainly comprises lowlands, hill dipterocarps and secondary forest, with an estimated 138 species of trees from 40 families. The Euphorbiaceae family is the most common flora family, whereas Dipterocarp trees are the most abundant tree species in the Park (Zainuddin et al. 2012). The Gunung Belumut Forest Eco Park is famous for recreational activities such as hiking, camping, picnicking and providing an escape for local people during holidays. The Park is easily accessible, being located in central Johor, approximately 32 km from the City of Kluang.

### Bird data collection

Prior to sampling, a 400 m x 200 m main plot, covering a total of eight hectares, was established in Kluang Forest Reserve. We randomly built four subplots, each measuring 50 m x 50 m, within the main plot to conduct concurrent sampling on small mammals and herpetofauna. We introduced multiple approaches, including mist netting, bird call recording and point count observation, for the assessment of bird richness and abundance. A total of four sampling sessions, each consisting of five days of trapping, were conducted from March to July 2022. For mist netting, we deployed a total of 10 mist nets using 5- and 3-m poles. All of the nets were placed within or near the sampling plot along potential bird flight paths with a minimum distance of 50 m between nets. The mist nets were opened for 5 consecutive days and they were checked every two hours from 8.00 a.m. to 12.00 p.m. and from 5.00 p.m. to 7.00 p.m. All of the birds caught in the net were carefully removed and placed inside a cloth bag. All of the birds were thoroughly examined and their morphological characteristics were measured to identify them at the species level. We followed the field guide by Robson (2014) for bird species identification.

Point count observation was conducted for 3 consecutive days along five 100-m line transects. Within each line transect, ten points were established for observation with a minimum 15 minutes duration. The line transects were established from the lower to a higher elevation (encompassing both lowland and hill forest areas) within the sampling plot. For each line transect, one person observed and counted the number of birds, while the other person recorded the observations on a data sheet and identified the species using a reference book. All transects were located at least 50 m apart to avoid double counting. The observations were aided by the use of a DSLR camera and binoculars.

Bird call was also used to identify species. All of the calls of birds heard in the study plot were recorded from the start of the call until the end using two smartphones. Multiple call recordings of the same bird were recorded to ensure the same species were detected. The calls were analysed using BirdNET software (Cornell Lab of Ornithology) (Kahl et al. 2021). The recordings from the smartphone were transferred to the servers for processing. BirdNET uses machine-learning for detecting and classifying avian sounds: an artificial network provides the most probable bird species in the recording, using a GPS service to form predictions based on location and date.

## Alpha and beta diversity analyses

Alpha and beta diversities were calculated using the vegan package (Oksanen et al. 2018) in R Studio Statistical Software (RStudio 2023). For alpha diversity, species richness, abundance, species rarefaction and extrapolation were analysed using the iNEXT package (Hsieh et al. 2023). For beta analysis, secondary data obtained from the forest reserves in Johor and presented in published papers were extracted and analysed. SIMPER analysis was used to identify significant differences in species amongst the forest reserves and principal coordinate analysis (PCoA) was performed to analyse dissimilarities amongst the forest reserves. We used SIMPER and Bray-Curtis analyses based on species richness, which inherently does not account for species abundance. This approach was chosen to mitigate the influence of variations in sampling effort and methods across the different studies. Given that species richness focuses solely on presence-absence data, the impact of sampling discrepancies on our comparisons should be minimal.

The Bray–Curtis dissimilarity and Jaccard distance indices were used to construct a distance matrix, based on the pairwise dissimilarities between samples in the measured PCoA. The Bray–Curtis dissimilarity index is a measure of the compositional dissimilarity between sample sets, which considers the presence and abundance of the species in a sample, but ignores the shared abundances. The Jaccard distance is a measure of the dissimilarity between sample sets that focus on the presence or absence of species; this index can be easily interpreted as a ratio of the number of species found in only one of the samples to the number of species found in either sample.

## Results

### Alpha diversity of avifauna assemblage in Gunung Belumut Eco Park

**Figure 2.**
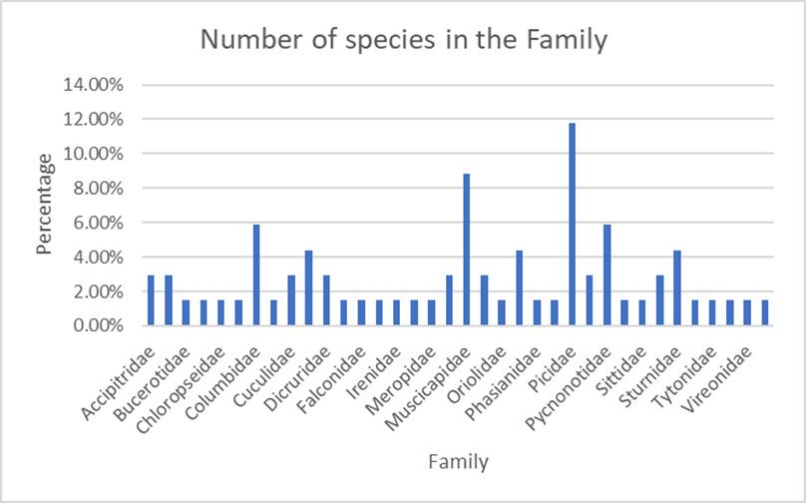
Total number of species in each family.

The survey conducted between March to July 2022 yielded records of 68 bird species spanning 36 families (Table 1). In Fig. 2, the largest number of species was recorded for the Picidae family, accounting for 11.76% of the total, followed by the Muscicapidae family at 8.82% and Pycnonotidae and Columbidae, both at 5.88%. Only one species each was recorded for 21 families, each representing 1.47% of the total. All the dataset information was deposited into Global Biodiversity Information Facility (GBIF)

**Figure 3.**
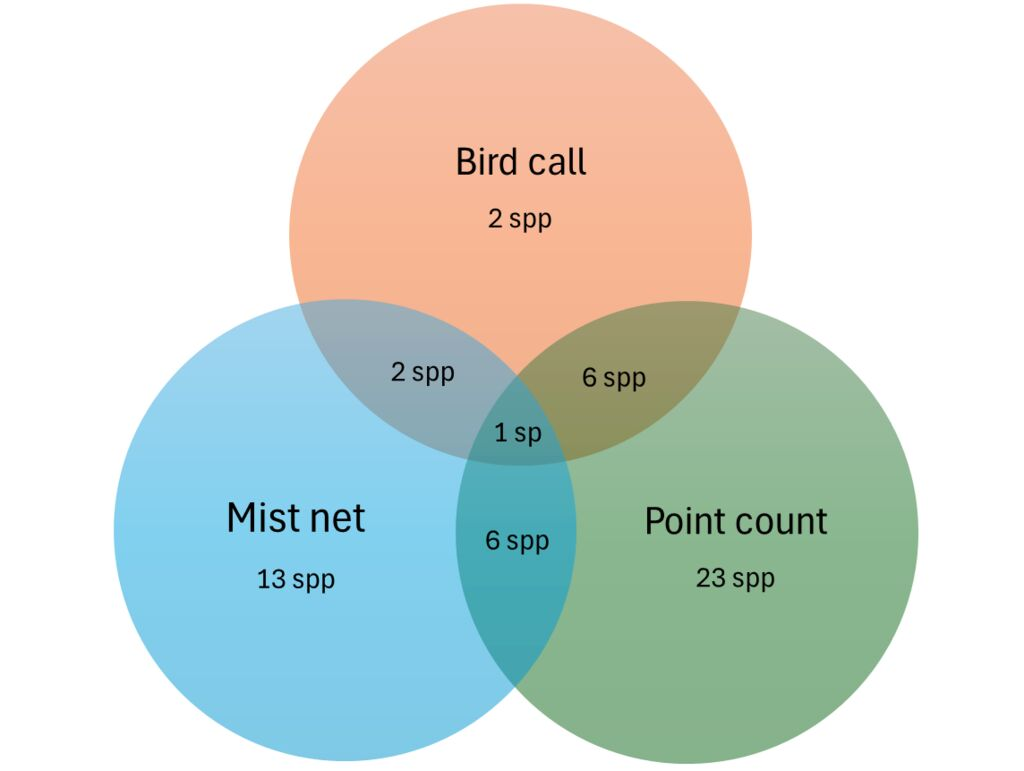
Number of bird species recorded in Kluang FR based on bird call, mist netting and point count observations.

A total of 250 mist-netting efforts were executed during the sampling sessions. As shown in Fig. 3, 58% of the bird species were identified through observation, 30% through mist netting and 12% from bird calls. During the sampling sessions, one species was consistently recorded using all three methods, eight species were documented using a combination of two methods and 59 species were identified using only one method. The sole species identified through all three sampling methods was the White-rumped Shama (*Kittacinclamalabarica*). Amongst the species captured using a mist net, the White-rumped Shama (*K.malabarica*) was the most abundant, with five individuals, followed by the Hairy-backed Bulbul (*Tricholestescriniger*) with four individuals. For twelve other species, we only captured a single individual.

**Figure 4.**
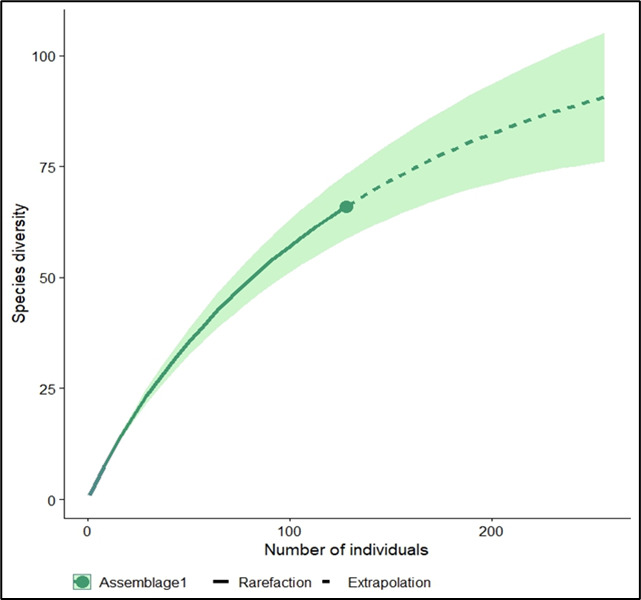
Species accumulation and extrapolation curve based on sample abundance.

**Figure 5.**
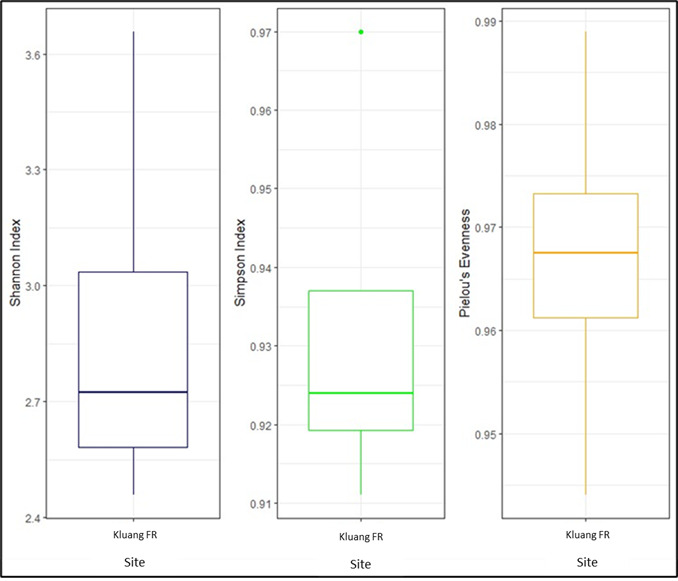
Diversity Index in Kluang Forest Research.

As shown by the IUCN status listed in Table 1, most of the bird species in Kluang FR were classified as species of least concern. Only one species was classified as endangered (EN), the Blue-winged Leafbird (*Chloropsiscochinchinensis*); four were are classified as vulnerable (VU): the Wreathed Hornbill (*Rhyticerosundulatus*), Brown-chested Jungle Flycatcher (*Cyornisbrunneatus*), Long-tailed Parakeet (*Belocercuslongicaudus*) and Javan Myna (*Acridotheresjavanicus*). According to the results of the Chao 1 species estimator, the estimated species richness was approximately 108,447 bird species in the Kluang FR. This finding suggested that approximately 63% of the bird assemblages were accounted for in this study based on the species accumulation curve (Fig. 4). The Shannon-Wienner Index indicated the value of bird diversity in Kluang FR ranged between 2.622 and 3.634; while the Simpson Index ranged between 0.922 and 0.9634 and the Pielou`s evenness index ranged between 0.966 and 0.989 (Fig. 5).

### Beta diversity

Secondary data on the birds in the five selected forest reserves in Johor were collected and analysed in conjunction with the data we obtained in this study. The five forest reserves were located within the State of Johor: Kluang Forest Reserve (KFR) (data from the current study and Shahrul Anuar et al. (2012)), Gunung Ledang (GL) (Adrus et al. 2013), Panti Forest Reserve (Panti FR) (Shahrul Anuar et al. 2009), Ulu Sedili Forest Reserve (US) (Alwani et al. 2019) and Ayer Hitam Utara (Ng and Norazlimi 2022).

In these studies, 204 bird species from 50 families were recorded. Panti FR had the largest number of species, at 179, followed by the KFR with 102 species, the US with 58 species, GL with 21 species and AHU with only 16. The Muscicapidae was the family with the most species (17), which was closely followed by the Pycnonotidae family with 15 species. For another 12 families, we only documented one species each. The White-rumped Shama (*K.balabarica*) was the only species found in all forest reserves; seven other species were recorded in four of the five forest reserves. Another 74 species were found in just one of the forest reserves.

**Figure 6.**
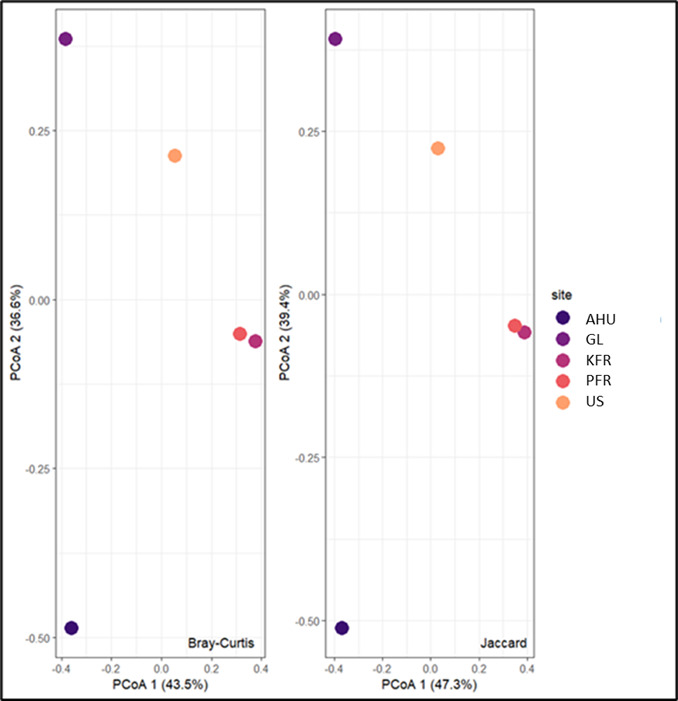
Principal Coordinates Analysis in different forest reserves; Kluang Forest Reserve (KFR), Gunung Ledang (GL), Panti Forest Reserve (PFR), Ulu Sedili Forest Reserve (US) and Ayer Hitam Utara (AHU).

According to the results of the SIMPER analysis, the species that contributed the most to the differences in the investigated assemblages was the White-bellied Woodpecker (*Dryocopusjavensis*), being 70% significantly different from the other species. The Jaccard and Bray–Curtis indices were used for principal coordinate analysis (PCoA) to measure the dissimilarity in the bird composition amongst the five forest reserves. Amongst these reserves, KFR and GL were the most dissimilar (J = 95.7%, B = 90%) and the KFR and Panti FR were the most similar (Fig. 6).

## Discussion

Species of the Picidae family were the most frequently observed in the Kluang FR. The prevalence of this woodpecker species could be attributed to the abundance of Dipterocarp trees and snags within the Kluang FR, which offer an ideal habitat for these birds. Woodpeckers prefer to nest within tall tree trunks and forage on the branches and trunks of dead or dying trees; one notable example of this is the White-bellied Woodpecker (*Dryocopusjavensis*) (Short 1978, Short 1982, Wells 1999, Winkler et al. 1995). Santharam (2003) reported that White-bellied Woodpeckers cannot survive in habitats lacking large dead or dying trees, which are essential for nesting; live small trees are used by foraging individuals.

Woodpeckers can harmoniously co-exist with other woodpecker species due to the differences in the foraging behavior of some of these species. They partition the available resources by foraging at different heights (Styring and Ickes 2003) and employing distinct strategies to reduce intraspecific conflict. In our study, the families for which we found a large number of species were those that dominate the understorey habitat of Malaysian forest reserves. This result is corroborated by those of studies conducted by Mansor and Sah (2012), Diau et al. (2013), Nor Hisham and Ramli (2013), Shafie et al. (2018) and Munian et al. (2023). According to Strange and Jeyarajasingam (1999), each bird family and species is adapted to a particular habitat for the purpose of feeding and breeding. As a result, most of the birds captured via mist netting belonged to species that typically perch on trees or remain closer to the ground in areas with more open spaces and shrubbery.

Notably, the mist net pole was not long enough to capture birds that inhabit the canopy level. The bird species list shows that most birds were identified through the point count method. This occurred because the sampling plots were surrounded by towering Dipterocarp trees with dense canopies. Some species primarily forage within the forest canopy and, thus, would rarely descend to the level of the nets, which reached only 3–5 m above the ground. Moreover, the results obtained through the point count method mainly featured bird species observed in higher and more open areas. However, the bird diversity and distribution patterns were similar for both methods. Andrew et al. (1997) and Enrique and Sandra (2005) found that different sets of common species with similar frequency were detected with these two methods, although many uncommon species were only detected with the point count method. Mist netting helps with detecting smaller, cryptic or secretive bird species, especially those in the understorey; large-bodied and rare species can be detected via point count observations. As such, different forest layers and bird types are factors that need to be considered when assessing bird diversity.

This study uncovered a notable inconsistency in the bird assemblages within the Kluang FR, which was driven by its species richness, resulting in numerous individuals being documented from a wide range of species. Amongst the species observed, the largest number of individuals was recorded for the White-rumped Shama (*K.malabarica*), being recorded using all three methods (mist netting, point counting and calls). This suggests that the White-rumped Shama was the most prevalent in the area. Additionally, the White-rumped Shama is insectivorous, primarily foraging on the ground and in low vegetation for arthropods, worms and berries (del Hoyo et al. 2005), explaining why we captured so many of this species in this study. The results of the Chao1 species estimator showed that 63% of the species in the Kluang FR were documented. The species accumulation curve showed that an asymptote was not reached, indicating that the sampling effort was insufficient, despite the different methods used in this study. The graph suggests the possibility of common and rare species that were not discovered during the sampling process. This shortfall may have resulted in under-representation of both rare and cryptic species that were less likely to be detected within the study period and sampling scope. Furthermore, variations in habitat complexity, seasonal fluctuations in species presence and limitations in sampling frequency and spatial coverage likely contributed to this under-sampling. To obtain a more accurate representation of the bird community in Kluang FR, we recommend expanding the sampling effort across multiple similar plots and considering additional sampling across different seasons. Such an approach would enhance our understanding of the actual bird assemblage and allow for a more comprehensive assessment of species richness within this ecologically important area.

The PCoA results showed that the difference between the Kluang FR and Gunung Ledang was the largest amongst the considered forest reserves. This result may be biased as it could have been influenced by the available published data for Gunung Ledang, which only included a species list from a rapid assessment, whereas the Kluang FR data were collected from a series of sampling sessions. Conversely, the Kluang and Panti FRs were the most similar. When searching for secondary data on birds in the Johor forest reserves, we found that relatively few published papers were available. Considering the variety of bird species amongst the five forest reserves, one might expect some forest reserves to host more species than others. For instance, Gunung Ledang comprises different types of vegetation, including lowland and high-altitude Dipterocarp, montane and Arecaceous forests (Kiew 1992). Given this diversity of habitats, a rich variety of bird species would be expected. However, the data available from Adrus et al. (2013) could not sufficiently and comprehensively represent the bird diversity in Gunung Ledang, as the data were obtained from a rapid assessment. A similar issue was encountered for the Ayer Hitam Utara Forest Reserve, being poorly explored by ecologists; so, the published data are limited. In contrast, the Panti Forest Reserve has received more attention and is more popular as a bird sanctuary in Johor, resulting in a wealth of available data. Normaisharah and Norazlimi (2019) also highlighted the lack of research on bird diversity in Johor beyond the Panti Forest Reserve.

The species checklist presented here is still far from exhaustive, yet it should be treated with caution, as it is likely to expand with additional sampling effort. The observed similarity between the study site and the five examined forest reserves may also be influenced by potential inconsistencies in sampling effort and methodology across sites. Despite these limitations, this information remains crucial for developing and strengthening the classification of Environmentally Sensitive Areas in Malaysia, providing a valuable foundation for effective conservation planning.

## Conclusions

In this study, we identified the bird species in the Kluang FR to determine the bird assemblages in the forest reserves in Johor. Different approaches for bird identification are recommended to increase the number and species of birds detected, including cryptic and rare bird species. Secondary data are crucial for any study; our results highlight the importance of documenting and publishing data, as well as the need to study more areas in Johor due to the lack of published species checklists in forest reserves. The information provided from this study will hopefully contribute to the development of a new classification guideline for ESAs in PFRs.

## Data Resources

The data underpinning the analysis reported in this paper are deposited at GBIF, the Global Biodiversity Information Facility, https://doi.org/10.15468/tb3snc.

## Figures and Tables

**Table 1.** List of bird species recorded in Belumut Forest Reserve. Distribution status for recorded avifauna: R = Resident, M = Migrant. IUCN Red List of Threatened Species: LC: Least Concern, NT: Near Threatened, VU: Vulnerable, EN: Endangered. Protection status based on Wildlife Conservation Act 2010 (WCA, 2010): TP: Totally Protected, P: Protected, NP: Not Protected

**Family**	**Common Name**	**Species Name**	**Status**	**IUCN Status**	**WCA**
Accipitridae	Changeable Hawk-eagle	*Nisaetuscirrhatus*	R	LC	TP
	Crested Serpent Eagle	*Spilornischeela*	R	LC	TP
Alcedinidae	Blue-banded Kingfisher	*Alcedopeninsulae*	R	NT	TP
	White-throated Kingfisher	*Halcyonsmyrnensis*	R	LC	TP
Bucerotidae	Wreathed Hornbill	*Rhyticerosundulatus*	R	VU	TP
Camphepagidae	Scarlet Minivet	*Pericrocotusflammeus*	R	LC	TP
Chloropseidae	Blue-winged Leafbird	*Chloropsiscochinchinensis*	R	EN	TP
Cisticolidae	Yellow-bellied Prinia	*Priniaflaviventris*	R	LC	TP
Columbidae	Emerald Dove	*Chalcophapsindica*	R	LC	P
	Green Imperial Pigeon	*Duculaaenea*	R	NT	TP
	Thick-billed Green Pigeon	*Treroncurvirostra*	R	LC	TP
	Little Green Pigeon	*Treronolax*	R	LC	P
Coraciidae	Dollarbird	*Eurystomusorientalis*	R&M	LC	TP
Cuculidae	Chestnut-breasted Malkoha	*Phaenicophaeuscurvirostris*	R	LC	TP
	Raffles Malkoha	*Rhinorthachlorophaea*	R	LC	TP
Dicaeidae	Orange-bellied flowerpecker	*Dicaeumtrigonostigma*	R	LC	TP
	Scarlet-breasted Flowerpecker	*Prionochilusthoracicus*	R	NT	TP
	Yellow-breasted Flowerpecker	*Prionochilusmaculatus*	R	LC	TP
Dicruridae	Ashy Drongo	*Dicrurusleucophaeus*	R	LC	TP
	Greater Racket-tailed Drongo	*Dicrurusparadiseus*	R	LC	TP
Eurylaimidae	Banded Broadbill	*Eurylaimusjavanicus*	R	NT	TP
Falconidae	Black-thighed Falconet	*Microhieraxfringillarius*	R	LC	TP
Hemiprocnidae	Whiskered Treeswift	*Hemiprocnecomata*	R	LC	TP
Irenidae	Asian Fairy-bluebird	*Irenapuella*	R	LC	TP
Megalaimidae	Red-throated Barbet	*Psilopogonmystacophanos*	R	NT	TP
Meropidae	Blue-throated Bee-eater	*Meropsviridis*	R	LC	TP
Monarchidae	Black-naped Monarch	*Hypothymisazurea*	R	LC	NP
	Asian Paradise Flycatcher	*Terpsiphoneparadisi*	R&M	LC	NP
Muscicapidae	Grey-chested Jungle-Flycatcher	*Cyornisumbratilis*	R	NT	TP
	Chestnut-naped Forktail	*Enicurusruficapillus*	R	NT	TP
	White-rumped Shama	*Kittacinclamalabarica*	R	LC	P
	Asian Brown Flycatcher	*Muscicapadauurica*	M	LC	TP
	Oriental Magpie Robin	*Copsychussaularis*	R	LC	P
	Brown-chested Jungle Flycatcher	*Cyornisbrunneatus*	M	VU	TP
Nectariniidae	Little Spiderhunter	*Arachnotheralongirostra*	R	LC	TP
	Purple-naped Sunbird	*Kurochkinegrammahypogrammica*	R	LC	TP
Oriolidae	Dark-throated Oriole	*Oriolusxanthonotus*	R	NT	TP
Pellorneidae	Moustached babbler	*Malacopteronmagnirostre*	R	LC	TP
	Black-capped Babbler	*Pellorneumnigrocapitatum*	R	LC	TP
	White-chested babbler	*Pellorneumrostratum*	R	NT	TP
Phasianidae	Red Junglefowl	*Gallusgallus*	R	LC	P
Phylloscopidae	Dusky Warbler	*Phylloscopusfuscatus*	M	LC	TP
Picidae	Greater Flameback	*Chrysocolaptesguttacristatus*	R	LC	NP
	Checker-throated Woodpecker	*Chrysophlegmamentale*	R	NT	TP
	Banded Woodpecker	*Chrysophlegmaminiaceum*	R	LC	TP
	Common Flameback	*Dinopiumjavanense*	R	LC	TP
	White-bellied Woodpecker	*Dryocopusjavensis*	R	LC	TP
	Grey-and-buff woodpecker	*Hemicircussordidus*	R	LC	TP
	Buff-necked Woodpecker	*Meiglyptestukki*	R	NT	TP
	Crimson-winged Woodpecker	*Picuspuniceus*	R	LC	TP
Psittacidae	Long-tailed Parakeet	*Belocercuslongicaudus*	R	VU	TP
	Blue-crowned hanging parrot	*Loriculusgalgulus*	R	LC	P
Pycnonotidae	Yellow-bellied Bulbul	*Alophoixusphaeocephalus*	R	LC	TP
	Red-eyed Bulbul	*Pycnonotusbrunneus*	R	LC	TP
	Cream-vented Bulbul	*Pycnonotussimplex*	R	LC	TP
	Hairy-backed bulbul	*Tricholestescriniger*	R	LC	TP
Rhipiduridae	Pied Fantail	*Rhipidurajavanica*	R	LC	TP
Sittidae	Velvet-fronted Nuthatch	*Sittafrontalis*	R	LC	TP
Strigidae	Collared Scops Owl	*Otuslettia*	R	LC	TP
	Barred Eagle-owl	*Bubosumatranus*	R	NT	TP
Sturnidae	Javan Myna	*Acridotheresjavanicus*	R	VU	NP
	Asian Glossy Starling	*Aplonispanayensis*	R	LC	NP
	Common Hill Myna	*Graculareligiosa*	R	LC	P
Trogonidae	Red-naped Trogon	*Harpacteskasumba*	R	NT	TP
Tytonidae	Barn Owl	*Tytoalba*	R	LC	TP
Vangidae	Rufous-winged Philentoma	*Philentomapyrhoptera*	R	LC	NP
Vireonidae	White-bellied erpornis	*Erporniszantholeuca*	R	LC	TP
Zosteropidae	Hume's White-eye	*Zosteropsauriventer*	R	LC	P
